# Nocardiosis: serie de casos y revisión bibliográfica

**DOI:** 10.7705/biomedica.7486

**Published:** 2025-05-30

**Authors:** José Camilo Álvarez-Rodríguez, Carlos A. Solórzano-Ramos, Viviana López-Ramírez, Luisa Torres-Rubio, Ana Ovalle-Gómez, Jersson Camilo Sánchez-Gámez, Cindy L. Beltrán-Endo, María J. López-Mora, Julio C. Gómez-Rincón, Cristian L. Cubides-Cruz, Rose M. Jaramillo-Calle, Vanessa Correa-Forero, Nidia Gabriela Cristina García, Sonia Isabel Cuervo-Maldonado

**Affiliations:** 1 Grupo de Infectología, Instituto Nacional de Cancerología, Bogotá, D. C., Colombia Instituto Nacional de Cancerología Instituto Nacional de Cancerología Bogotá, D. C. Colombia; 2 Grupo de Investigación Enfermedades Infecciosas en Cáncer y Alteraciones Hematológicas (GREICAH), Instituto Nacional de Cancerología, Bogotá, D. C., Colombia Instituto Nacional de Cancerología Instituto Nacional de Cancerología Bogotá, D. C. Colombia; 3 Servicios de Medicina Interna e Infectología, Hospital Universitario Clínica San Rafael, Bogotá, D. C., Colombia Hospital Universitario Clínica San Rafael Hospital Universitario Clínica San Rafael Bogotá, D. C. Colombia; 4 Servicio de Medicina Interna, Clínica Centenario, Bogotá, D. C., Colombia Clínica Centenario Clínica Centenario Bogotá, D. C. Colombia; 5 Servicio de Medicina Interna, Fundación Santa Fe de Bogotá, Bogotá, D. C., Colombia Fundación Santa Fe de Bogotá Fundación Santa Fe de Bogotá Bogotá, D. C. Colombia; 6 Facultad de Medicina, Universidad Surcolombiana, Neiva, Colombia Universidad Surcolombiana Universidad Surcolombiana Neiva Colombia; 7 Grupo de Infectología, Hospital San Ignacio, Bogotá, D. C., Colombia Hospital San Ignacio Hospital San Ignacio Bogotá, D. C. Colombia; 8 Servicio de Infectología, Fundación Centro de Tratamiento e Investigación sobre Cáncer (CTIC), Bogotá, D. C., Colombia Fundación Centro de Tratamiento e Investigación sobre Cáncer (CTIC) Fundación Centro de Tratamiento e Investigación sobre Cáncer (CTIC) Bogotá, D. C. Colombia; 9 Grupo de Investigación GIGA, Centro de Tratamientos e Investigación sobre Cáncer, Universidad El Bosque, Bogotá, D. C., Colombia Universidad El Bosque Universidad El Bosque Bogotá, D. C. Colombia; 10 Facultad de Medicina, Universidad El Bosque, Bogotá, D. C., Colombia Universidad El Bosque Universidad El Bosque Bogotá, D. C. Colombia; 11 Facultad de Medicina, Universidad Nacional de Colombia, Bogotá, D. C., Colombia Universidad Nacional de Colombia Universidad Nacional de Colombia Bogotá, D. C. Colombia

**Keywords:** nocardiosis, cavitación, absceso encefálico, tolerancia inmunológica, *Nocardia* infections, cavitary, brain abscess, immune tolerance

## Abstract

La nocardiosis es una infección causada por un bacilo grampositivo filamentoso que afecta en mayor medida a los pacientes inmunosuprimidos. Sus manifestaciones pueden ser localizadas o sistémicas. Para seleccionar el tratamiento, se debe considerar el órgano comprometido, la gravedad de la infección y el perfil de sensibilidad microbiana.

Se presentan 14 casos de pacientes con infección por *Nocardia* spp. atendidos en diferentes instituciones de salud de Bogotá entre enero del 2008 y noviembre del 2023. La información se obtuvo del laboratorio de microbiología, y se buscaron variables demográficas, clínicas y de laboratorio.

La edad promedio de los pacientes incluidos fue de 49,8 años (30 a 72 años), 10 eran hombres y nueve tenían un diagnóstico que implicaba inmunosupresión: seis tenían cáncer y tres, otras condiciones de inmunosupresión. Las comorbilidades más frecuentes fueron diabetes e hipertensión arterial sistémica. La presentación clínica de la nocardiosis fue crónica en 10 de los casos, y los órganos comprometidos fueron cerebro y pulmón en 7. Fue posible identificar la especie de *Nocardia* en cinco de los pacientes; uno de los evaluados presentó infección por *Cryptococcus* spp. El tratamiento para la nocardiosis fue prolongado e incluyó trimetoprim-sulfametoxazol en 12 casos; fallecieron cuatro pacientes. La infección por *Nocardia* spp. es principalmente oportunista, con aspectos clínicos y microbiológicos únicos. Es un diagnóstico diferencial de abscesos localizados o sistémicos en pacientes inmunosuprimidos. La sospecha clínica permite un enfoque cuidadoso en términos de diagnóstico y el inicio de tratamiento antibiótico empírico, que sigue siendo heterogéneo, puede tener un impacto positivo en la supervivencia.

## Abstract (en)

Nocardiosis is caused by a branched Gram-positive bacillus that affects mostly immunosuppressed patients. The clinical manifestations can be localized or disseminated, and the treatment depends on the affected organs, the severity of the disease, and the susceptibility profile.

Nocardiosis should be considered in the differential diagnosis of localized or disseminated abscesses in immunosuppressed patients. Early clinical suspicion would allow the start of empirical treatment, prompt microbiological research, and may positively impact survival. We present a case series of 14 patients with infection by *Nocardia* spp. who attended different health institutions in Bogotá between January 2008 and November 2023. The clinical microbiology laboratory provided information on demographic, clinical, and laboratory variables.

Fourteen patients were analyzed, with an average age of 49.8 years (30 to 72 years); 10 were men, and nine had a diagnosis involving immunosuppression: six had cancer and three had other underlying immunosuppression. Diabetes and hypertension were the most common comorbidities. The infection was chronic in 10 patients; the organs involved were the brain and lungs in 7 cases. *Nocardia* species’ was identified in five subjects; one of the patients presented infection with *Cryptococcus* spp. Nocardiosis treatment was prolonged and included trimethoprim-sulfamethoxazole in 12 cases; four patients died.

Infection by *Nocardia* spp. is primarily opportunistic, presents unique clinical and microbiological aspects, and is a differential diagnosis of localized or systemic abscesses in immunosuppressed patients. Clinical suspicion allows a careful approach in terms of diagnosis, and initiation of empiric antibiotic treatment can positively impact survival.

La nocardiosis es una infección causada por una bacteria grampositiva, filamentosa, débilmente ácido-alcohol resistente, aerobia, de crecimiento lento, ubicua en el ambiente y perteneciente al orden *Actinomycetales*. El reconocer la especie en los laboratorios clínicos es complejo ya que, con la evolución de la taxonomía y los métodos diagnósticos, cada vez se identifican nuevas especies que luego son incluidas en “complejos” según sus propiedades bioquímicas y la sensibilidad a antimicrobianos. Actualmente, se conocen más de 80 especies que pueden producir la enfermedad en el humano, la mayoría por los complejos *Nocardia nova*, *N. abscessus*, *N. transvalensis*, *N. farcinica*, *N. asteroides* tipo VI (*N. cyriacigeorgica*), *N. brevicatena*-*N. paucivorans* y *N. brasiliensis*^1^. *Nocardia* se transmite por ingestión, inoculación o inhalación, siendo esta última la ruta más común. Las manifestaciones clínicas se han clasificado anatómicamente como pulmonar, del sistema nervioso central, cutánea o diseminada ^1^.

Del 60 al 70 % de los casos de nocardiosis se presenta en pacientes con inmunodeficiencias, principalmente secundarias ^2^, con manifestaciones clínicas diversas, ya sea por lesiones focales o diseminadas, y cerca de un tercio de los casos se han descrito en pacientes inmunocompetentes. La infección pulmonar suele tener un factor predisponente en pacientes con enfermedades pulmonares estructurales preexistentes. Se ha reportado compromiso cutáneo, pulmonar y del sistema nervioso central, y colecciones intraabdominales ^3^^,^^4^. La formación de abscesos es el signo distintivo de la enfermedad. La inmunidad es principalmente de tipo celular (macrófagos y células T), pero cepas más virulentas de *N. asteroides* pueden resistir la acción mediada por los neutrófilos y otras pueden inhibir la fusión del fagosoma y el lisosoma, lo cual permite la supervivencia de formas latentes que, potencialmente, pueden causar infecciones graves y recaídas ^1^.

En este estudio, se presentan 14 casos de infecciones graves por *Nocardia* spp., seis de ellos con cáncer y cinco con otras enfermedades de base, todos con algún grado de inmunosupresión adquirida por causa del mismo cáncer, la exposición a quimioterapia o radioterapia u otra condición clínica; tres pacientes no reportaron ningún antecedente médico relevante. En todos los casos, se aisló el microorganismo mediante cultivo y, en cinco de ellos, se identificó la especie por espectrometría de masas por desorción-ionización láser asistida por matriz y análisis de tiempo de vuelo (MALDI-TOF). Se detectaron tres aislamientos de *N. farcinica*, uno de *N. cyriacigeorgica* y uno de *N. araonensis*.

El tratamiento definitivo incluyó trimetoprim-sulfametoxazol en 12 casos. Otros antibióticos utilizados fueron meropenem, ceftriaxona, amikacina y amoxicilina con ácido clavulánico. El tiempo de tratamiento fue prolongado en todos los casos y llegó, incluso, hasta 64 semanas. Cuatro de los pacientes fallecieron.

## Presentación de casos

En el cuadro 1, se resumen las principales características de los 14 pacientes incluidos, con diagnóstico de nocardiosis, atendidos en varias instituciones de Bogotá entre el 2008 y el 2023. La edad promedio de los sujetos fue de 49,8 años (30 a 72 años), nueve eran hombres y la ocupación fue conductor en cuatro; seis pacientes tenían diagnóstico de cáncer y, ocho, otras enfermedades de base. La presentación de nocardiosis fue crónica en ocho casos; los órganos más comprometidos fueron cerebro y pulmón, cada uno en ocho. En cinco pacientes se identificó la especie infecciosa. El tratamiento fue prolongado y doce de los casos fueron tratados con trimetoprim-sulfametoxazol. Fallecieron cuatro pacientes.

### Consideraciones éticas

El estudio cuenta con el aval de los comités de ética de las instituciones en las que fueron atendidos los pacientes y, en casos particulares, con el consentimiento informado de dos pacientes para la publicación de su caso.

## Discusión

*Nocardia* spp. cuenta con más de 80 especies patógenas, hace parte del orden Actinomycetales y del suborden Corynebacterineae. Su pared celular es de tipo IV ya que contiene ácido mesodiaminopimélico, arabinosa y ácido micólico, componentes que contribuyen a su virulencia ^5^. Esta bacteria es ubicua, se encuentra en el suelo, en la materia orgánica y en el agua.

La nocardiosis es una enfermedad de distribución mundial ^1^, anualmente se notifican entre 500 y 1.000 casos en los Estados Unidos ^1^ y, entre 90 y 130, en Italia ^1^. También, se han documentado casos en países ubicados en el trópico, como India, Pakistán e Irán, y en otros países, como Canadá, España y Australia ^1^. La incidencia de nocardiosis parece crecer cada año por el aumento de la población inmunocomprometida, el mejor reconocimiento de la enfermedad y la tecnificación de los métodos diagnósticos. En regiones tropicales como Centroamérica y Suramérica, la especie más frecuente es *N. brasiliensis*, responsable del 80 % de las infecciones cutáneas (micetomas), mientras que las especies *N. nova*, *N. farcinica* y *N. cyacigeorgica* se han asociado con infecciones sistémicas con compromiso pulmonar ^4^.

La edad de presentación depende de varios factores, como el estado inmunológico y la exposición a fuentes de infección. En esta serie, la edad promedio (49,8 años) fue ligeramente menor en comparación con la de unos estudios retrospectivos realizados en Michigan (55 ± 17 años) y Madrid (56 años) ^6^^,^^7^.

La nocardiosis rara vez afecta a personas sanas: del 60 al 70 % de los casos se presenta en pacientes con inmunosupresión primaria o secundaria (trasplante de órgano, neoplasia hematológica, sida, alcoholismo, diabetes, uso crónico de esteroides o exposición a inhibidores del TNF-α) ^5^. La mayoría de las veces, su presentación es diseminada ^7^. En pacientes con alteración del parénquima pulmonar (por ejemplo, enfermedad obstructiva crónica pulmonar, EPOC) ^8^, la vía aérea puede ser la puerta de entrada, como se sospecha en los casos uno y trece, quienes tenían compromiso pulmonar de base ^1^.

**Cuadro 1:** Descripción clínica de los pacientes con nocardiosis

No.	Edad	Sexo	Año*	Comorbilidad	Ocupación	Síntomas de ingreso	Duración	ELISA HIV	Órganos comprometidos	Hallazgos en imágenes diagnósticas	Muestra para cultivo de bacilos grampositivos	Especie/tiempo de crecimiento del cultivo	Terapia definitiva	Desenlace
1	70	H	2016	Cáncer basocelular bronquiectasias	Agricultor	Abscesos cutáneos, fiebre	3 meses	Neg.	Piel, pulmón, cerebro, abdomen	Abscesos cerebrales, musculocutáneos, peritoneales y pulmonares	Líquido de drenaje del absceso cutáneo	*Nocardia* spp.; 1 semana	Ceftriaxona, trimetoprim- sulfametoxazol	Fallecido
2	53	M	2019	Cáncer de cérvix	Administra una miscelánea	Fiebre	15 días	Neg.	Cerebro	Abscesos en ambos hemisferios cerebrales y córtico-subcorticales, en cerebelo y tallo	Sangre	*Nocardia* spp.	Trimetoprim-sulfametoxazol (13 días), meropenem (21 días)	Fallecido
3	30	M	2019	Cáncer de colon	Psicóloga	Fiebre	15 días	Neg.	Vasos sanguíneos	Sin abscesos	Sangre	*Nocardia* spp.; 4 días	Trimetoprim-sulfametoxazol (24 días), meropenem (21 días)	Fallecido
4	62	H	2020	Cáncer de próstata	Minero	Disnea, fiebre		Neg.	Pulmón	Nódulos múltiples y derrame pleural	Líquido pleural	*Nocardia* farcinica	Trimetoprim-sulfametoxazol (23 días intrahospitalarios, 6 meses de tratamiento ambulatorio), meropenem (13 días)	Vivo
5	46	H	2021	Leucemia linfoblástica aguda con trasplante haploidéntico de progenitores hematopoyéticos	Conductor	Convulsión	1 día	Neg.	Pulmón, hígado, cerebro y espacio masticador izquierdo	Abscesos cerebrales y un nódulo pulmonar; absceso en el espacio masticador izquierdo y lesiones hepáticas	Absceso del espacio masticador izquierdo	*Nocardia* farcinica	Meropenem (6 semanas), trimetoprim: sulfametoxazol (8 meses), amoxicilina- clavulanato (6 meses y medio)	Vivo
6	37	H	2018	Carcinoma neuroendocrino de células pequeñas	Perforador de túneles	Dolor torácico y diarrea	2 meses	Neg.	Pulmón	Absceso y cavitación pulmonar	Absceso	*Nocardia* cyriacigeorgica	Amikacina (19 días), meropenem (58 meses y 6 días) Tigeciclina (58 meses y 3 días)	Fallecido
7	35	M	2018	VIH (Carga viral de 54.890 copias, 5 linfocitos CD4 por μΙ)	Secretaria	Tos y disnea	3 meses	Pos.	Pulmón	Consolidaciones	Esputo	*Nocardia* araoensis	Meropenem y amikacina (21 días)	Vivo
8	34	H	2016	No identificado	Plomero	Celulitis y absceso	9 meses	Neg.	Piel	Sin abscesos La tomografía axial computarizada de tórax de diciembre del 2016 reveló nódulos pulmonares inespecíficos y granuloma calcificado del lóbulo inferior izquierdo; en diciembre, se identificó enfermedad de rodilla correspondiente a micetoma. La resonancia magnética mostró una extensa alteración de la señal de los tejidos blandos del aspecto medial de la rodilla por un proceso inflamatorio o neoplásico entre los diagnósticos diferenciales.	Absceso	*Nocardia* spp.	Meropenem y trimetoprim/ sulfametoxazol (3 meses) Amikacina (800 mg, vía intravenosa, cada 24 horas por 3 semanas) trimetoprim-sulfametoxazol (hasta por 12 meses)	Vivo
9	36	M	2016	Síndrome de Sjogren, uso crónico de esteroides	Operaria telefónica	Lesiones cutáneas y tos de evolución crónica	15 días	Neg.	Piel, cerebro y pulmón	Abscesos	Absceso cutáneo y líquido de lavado broncoalveolar	*Nocardia* spp.	Linezolid, meropenem, trimetoprim- sulfametoxazol (15 mg/kg) Amoxicilina-ácido clavulánico (1 g cada 12 horas por 12 meses)	Vivo
10	61	H	2008	Sin identificación	Sin dato	Cefalea y hemiparesia	15 días	Neg.	Cerebro	Absceso	Sangre	*Nocardia* spp.	Resección quirúrgica; ceftriaxona- metronidazol (3 días) trimetoprim- sulfametoxazol (15 mg/kg/día, vía intravenosa, por X días; luego, 5 tabletas al día por 4 semanas y, después, 4 tabletas al día hasta completar 2 meses, sin mejoría); ceftriaxona y amikacina (1 mes)	Vivo
11	62	H	2019	Enfermedad periodontal	Conductor	Inflamación en la pierna derecha	5 meses	SD	Piel	Osteomielitis en el tercio proximal y medio Absceso de la diáfisis femoral derecha, asociada a miositis; absceso dentro del compartimiento posterior del muslo con formación de trayecto fistuloso a la piel de la cara posteromedial del tercio proximal del muslo	Absceso	*Nocardia* spp.	Ampicilina sulbactam (3 g, vía intravenosa, cada 4 h por 42 días; en una nueva hospitalización se administró otro esquema de ampicilina sulbactam (3 g, vía intravenosa, cada 6 h por 56 días Se realizó lavado quirúrgico (15/06/19) y toma de cultivos. Secuestrectomía, desbridamiento y rimado endovascular del fémur derecho	Vivo
12	39	H	2008	Ninguno	Conductor de tractomula	Hemiparesia	1 día	Neg.	Cerebro	Absceso cerebral	Absceso cerebral (2 días: (probable; 4 días; confirmado)	*Nocardia* spp.	Resección quirúrgica; ceftriaxona y metronidazol (4 días); ceftriaxona y amikacina (6 semanas) y trimetoprim-sulfametoxazol (8 meses)	Vivo
13	61	H	2010	Fibroantracosis pulmonar (tuberculosis pulmonar tratada), hipotiroidismo, tabaquismo y consumo de alcohol Se documenta ¡nmunodeficiencia celular primaria y déficit de linfocitos CD4.	Conductor de taxi	Hemiparesia	1 día	Neg.	Cerebro y pulmón	Absceso cerebral	Sangre	*Nocardia* spp. en cerebro; criptococosis diseminada	Metronidazol (12 días), ceftriaxona (2 g cada 12 h por 6 semanas), meropenem (2 g cada 6 h por 14 días) Al terminar, ceftriaxona, trimetoprimsulfametoxazol (160 mg/800 mg cada 6 horas hasta completar 1 año) Resección quirúrgica	Vivo
14	72	M	2023	Diabetes mellitus de tipo 2 e hipertensión arterial	Cesante, (antes trabajaba en servicios generales	Disartria, desviación de la comisura labial hacia el lado izquierdo y parestesias en la hemicara izquierda	13 días	Neg.	Cerebro y pulmón	Masa cerebral frontal derecha y nodulo pulmonar en el lóbulo superior derecho	Absceso cerebral	*Nocardia farcinica; 48 horas*	Resección quirúrgica de la lesión cerebral; (meropenem 1 g cada 8 h por 3 semanas), trimetoprimsulfametoxazol (160 mg/800 mg, tabletas vías oral, cada 8 h por 3 semanas). Al terminar, amoxicilina-. clavulanato y trimetoprimsulfametoxazol (3 meses)	Vivo

Neg.: negativo; Pos.: Positivo; SD: sin dato

* Año de consulta

En un estudio europeo multicéntrico, se encontraron cinco factores de riesgo principales para nocardiosis en casos de trasplante de órgano sólido: edad del paciente, aumento de la concentración del inhibidor de la calcineurina un mes antes del diagnóstico, uso de tacrolimus o corticosteroides en el momento del diagnóstico y duración de la estancia en la unidad de cuidados intensivos después del trasplante ^1^.

La nocardiosis pulmonar y diseminada es más prevalente en sujetos inmunosuprimidos, mientras que las manifestaciones cutáneas son más frecuentes en personas inmunocompetentes. En la presente selección de casos, nueve se encontraban en estado de inmunosupresión; de estos, seis padecían alguna enfermedad neoplásica en órganos sólidos (piel, cérvix y próstata). También, se registró inmunosupresión por disminución del número de linfocitos CD4 (infección por HIV e inmunodeficiencia primaria). La frecuencia de inmunosupresión en esta serie coincide con la de reportes de la literatura en los que la nocardiosis se asocia con inmunosupresión en más del 60 % de los pacientes ^8^^,^^9^.

En diferentes informes de series sobre pacientes positivos para HIV positivo, la nocardiosis ocurre del 3,4 al 16,7 % ^1^ de los casos. En este grupo de pacientes, la enfermedad se puede manifestar como localizada o diseminada. Cuando el recuento de linfocitos CD4^+^ es menor de 50 células/ μl y en caso de encontrarse compromiso pulmonar o pericárdico, se debe sospechar la nocardiosis y, aunque se ha informado ocasionalmente, se debe considerar la coinfección con *Mycobacterium tuberculosis* y micobacterias no tuberculosas ^1^.

La enfermedad se ha reportado en pacientes con cáncer; Wang *et al*. ^10^ describieron 132 pacientes con nocardiosis entre el 2002 y el 2012 en los Estados Unidos. Además, se ha descrito en pacientes con enfermedades autoinmunitarias y con trasplante de progenitores hematopoyéticos, como en la serie de Bambace *et al*., en la que de 440 casos de trasplante de progenitores hematopoyéticos, entre el 2007 y el 2011, once cursaron con nocardiosis ^11^. Igualmente, la enfermedad se ha documentado en pacientes con síndrome nefrótico y uso previo de corticoides ^1^.

Minero *et al*. ^7^ evaluaron, entre 1995 y 2009, 38 casos de nocardiosis en un hospital de referencia de Madrid. Los autores encontraron que los defectos de la inmunidad estaban presentes en la mayoría de las formas diseminadas ^12^.

Kim *et al*. estudiaron una serie de 54 casos de nocardiosis confirmados por cultivo y recolectados en un lapso de 13 años en la República de Corea. Los autores describieron las características de la enfermedad según el estado inmunológico: al comparar el 33 % de los pacientes inmunocompetentes con el grupo de los inmunocomprometidos, no encontraron diferencias estadísticamente significativas respecto a las características clínicas, radiológicas o microbiológicas, excepto que la cavitación pulmonar y la coinfección fueron más frecuentes en los inmunosuprimidos. El análisis de los sitios de infección mostró resultados similares a los de la presente serie; el 83 % de los pacientes tenía compromiso pulmonar y, en un tercio de estos, la nocardiosis fue diseminada; el compromiso del sistema nervioso central se observó en el 63 % de los casos ^13^.

De igual manera, Wang *et al*. ^10^ describieron 132 casos de nocardiosis en pacientes con cáncer en un periodo de 12 años: el 54,5 % con neoplasia maligna hematológica y el 43,3 %, con cáncer de órgano sólido. Las especies de *Nocardia* más frecuentes fueron *N. nova*, *N. cyriacigeorgica*, *N. farcinica* y *N. abscesus*, y tenían sensibilidad in vitro a trimetoprim-sulfametoxazol, linezolid, amikacina y claritromicina.

En el 80 % de los pacientes con neoplasia maligna hematológica, se encontró linfopenia y hubo uso de esteroides; el 100 % presentó compromiso pulmonar, y el 60 % tuvo manifestaciones clínicas de enfermedad diseminada en piel, ganglios linfáticos y sistema nervioso central ^14^. El promedio de supervivencia fue de 65 días. Se ha descrito el impacto negativo en la supervivencia de quienes cursan con alguna enfermedad sistémica, tienen abscesos cerebrales, están inmunosuprimidos o son mayores de 55 años, cuya mortalidad es cercana al 50 % (15). En la serie aquí presentada, cuatro pacientes fallecieron durante el tratamiento.

### Presentación clínica

**Figura 1:**
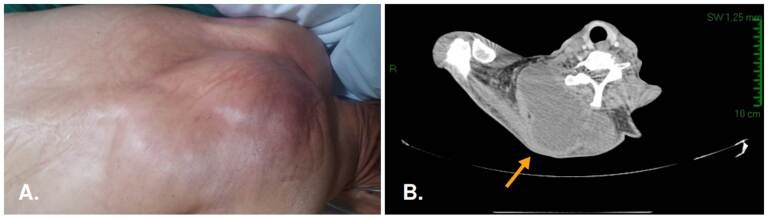
A. Caso 1. Lesión ubicada en el dorso del hemitórax derecho, aproximadamente de 20 cm de diámetro; B. Múltiples lesiones musculares, de tipo colección en planos, de la región cervical dorsal y estructuras del manguito rotador derecho.

**Figura 2:**
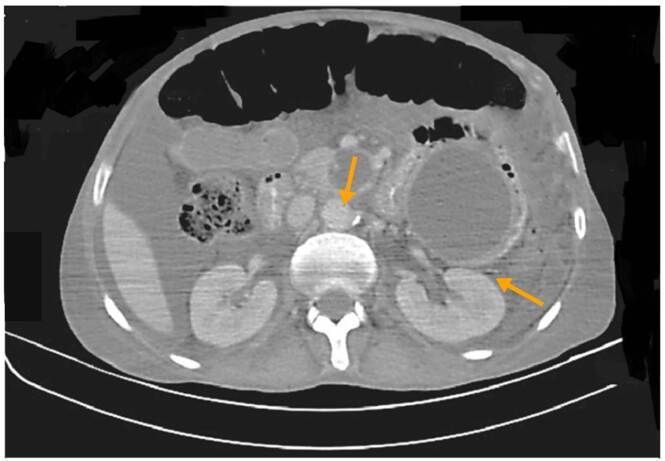
Caso 1. Tomografía axial computarizada de abdomen. Se observa una lesión hipodensa localizada en el cuerpo del páncreas, de morfología lobulada y con diámetro de más de 66 mm x 34 mm; además, tres lesiones hipodensas nodulares: una relacionada con el proceso uncinado del páncreas (23 mm) y dos localizadas entre las asas intestinales del flanco izquierdo (57 mm y 25 mm).

**Figura 3:**
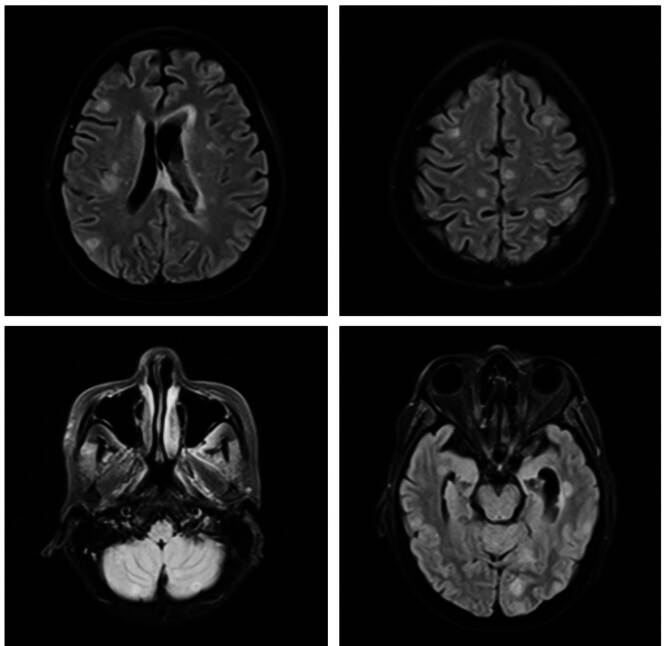
Caso 3. Resonancia magnética nuclear cerebral con contraste. Se observan múltiples lesiones intraaxiales, de localización córtico-subcortical, que comprometen ambos hemisferios cerebrales, los núcleos grises de la base, el cerebelo y el plexo coroideo en el interior del ventrículo lateral izquierdo. Por sus características y el contexto clínico de la paciente, las lesiones son indicativas de microabscesos secundarios a nocardiosis.

**Figura 4:**
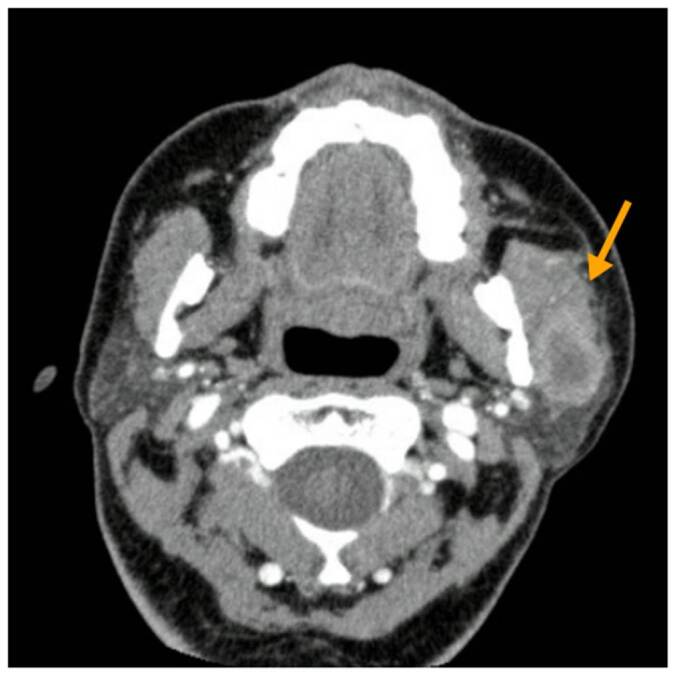
Caso 5. Tomografía axial computarizada de cara con contraste. Se observa una masa en el espacio masticador izquierdo indicativa de un absceso.

**Figura 5:**
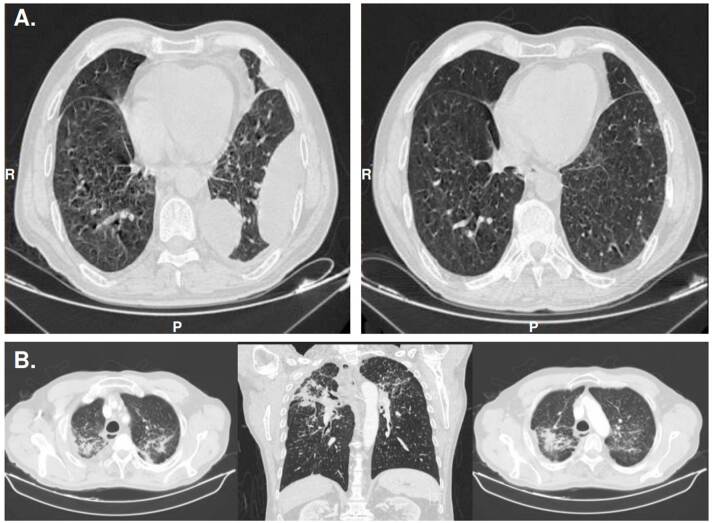
Caso 4. Compromiso pleuropulmonar. A. Derrame pleural tabicado localizado en el tercio proximal de la cisura mayor izquierda, de 22 mm x 20 mm, y en los segmentos posteriores del lóbulo inferior izquierdo, con un espesor mayor de 46 mm. Se observa una distorsión de la arquitectura de los ápices pulmonares asociada a consolidaciones que ejercen tracción sobre los hilios pulmonares. Se evidencian múltiples micronódulos de distribución multisegmentaria que indican el diagnóstico diferencial de silicosis conglomerada ganglionar. B. La tomografía computarizada de tórax con alta resolución (TACAR) revela resolución del derrame pleural, engrosamiento pleural basal izquierdo y opacidades con patrón de vidrio esmerilado.

El signo distintivo de la nocardiosis es la formación de abscesos ^1^, como se evidencia en las figuras 1-4. Usualmente, la progresión es crónica, independientemente de las barreras anatómicas, con tendencia a recurrir o recaer a pesar de recibir el tratamiento adecuado. Como se mencionó, los afectados suelen estar inmunocomprometidos; el inicio es insidioso y el curso clínico indolente; más de la mitad de los pacientes tienen compromiso pleural (figura 5A) y pulmonar (figura 5B).

La nocardiosis pulmonar puede manifestarse como neumonía, masa inflamatoria endobronquial, absceso pulmonar o enfermedad cavitaria con extensión a estructuras contiguas superficiales y profundas. El cerebro, los huesos, la piel, los ojos, el corazón, las articulaciones y los riñones son los sitios extrapulmonares más comunes, y el sistema nervioso central es el más comúnmente involucrado ^1^.

La diseminación hematógena puede causar bacteriemia y afectación de cualquier órgano, especialmente, del sistema nervioso central (absceso cerebral o meningitis) (figura 3), riñones, huesos y articulaciones. En las infecciones sistémicas, la nocardiosis se comporta como una bacteria piógena típica, capaz de inducir bacteriemia ^16^.

*Nocardia* es ubicua en el suelo, por lo que la inoculación cutánea es común. Puede provocar celulitis, pioderma y formación de abscesos que se asemejan a los generados por *Staphylococcus* o *Streptococcus*. La infección puede propagarse de los vasos linfáticos a los ganglios linfáticos regionales, dando lugar a la nocardiosis linfocutánea, un cuadro clínico que puede confundirse con la esporotricosis causada por *Sporothrix schenckii*. A partir de una lesión localizada, *Nocardia* puede producir micetomas en pies, piernas, brazos, manos o cualquier otro sitio del cuerpo. *Nocardia brasiliensis* es la especie más frecuentemente implicada en la presentación esporotricoide, mientras que las infecciones cutáneas debidas a *N. asteroides* son, en su mayoría, de resolución espontánea ^16^.

En algunos casos, se han descrito infecciones por *Nocardia* asociadas con la atención en salud, ya sea por inoculación directa, ingestión o inhalación de la bacteria presente en el polvo del ambiente hospitalario o en alimentos, agua o instrumentos médicos contaminados ^1^. Por ejemplo, *N. asteroides* se relacionó con un brote en seis pacientes en una unidad de trasplante renal ^1^. *Nocardia farcinica* se ha encontrado en heridas quirúrgicas esternales, en las manos de un trabajador de la salud y en infecciones asociadas con el catéter venoso central con formación de biopelícula ^1^.

Cuando la manifestación de la nocardiosis es pulmonar, suele haber una alteración previa del parénquima pulmonar (bronquiectasias y enfermedad pulmonar crónica, entre otras). En estos pacientes, es muy probable que la vía aérea hubiese sido la puerta de entrada del microorganismo mediante su inhalación del medio ambiente ^12^^,^^14^. Esto pudo suceder en la mayoría (60 a 81 %) de los pacientes que tuvieron compromiso pulmonar ^5^.

Cabe resaltar que, en Colombia, se han publicado algunos casos de nocardiosis cerebral, especialmente con formación de absceso, pero también, hubo un caso de meningitis primaria por *Nocardia* spp.; estos casos se presentaron tanto en inmunosuprimidos como en inmunocompetentes ^15^^,^^17^.

La nocardiosis diseminada se presenta con compromiso multiorgánico y gran morbimortalidad; el 44 % de los pacientes desarrolló abscesos cerebrales y granulomas. Sin embargo, otros órganos blanco son hueso, riñón, retina y corazón. Se describen otras manifestaciones menos frecuentes, como endocarditis en válvula nativa, artritis, absceso testicular, peritonitis, meningoencefalitis y masas intraabdominales ^18^. La enfermedad diseminada se presentó en seis casos de esta serie ^19^.

En dos de los seis casos con cáncer presentados aquí, se identificó *N. farcinica* como especie infecciosa. Sin embargo, los laboratorios clínicos institucionales donde se procesaron las muestras de los pacientes utilizan sistemas automatizados de microbiología, tipo Vitek™ (BioMérieux) o Phoenix™(Becton Dickinson), que solo permiten identificar el género. No se tuvo acceso a paneles para practicar las pruebas de sensibilidad *in vitro*. Solo en dos casos se pudo identificar la especie (*N. farcinica*), por medio del estudio de proteómica con MALDI-TOF. Esta es la razón por la que el tratamiento antimicrobiano administrado a los pacientes se ajustó según lo descrito en la literatura. Es importante anotar que cinco de los pacientes presentados en esta serie tenían cáncer de órgano sólido y solo uno tenía una neoplasia hematológica maligna, quien, a pesar de estar en profilaxis con trimetoprim-sulfametoxazol, presentó nocardiosis diseminada.

### Diagnóstico

El diagnóstico de la nocardiosis es complejo; se basa en la identificación de la bacteria en el laboratorio y, para su confirmación, se necesitan exámenes microbiológicos e histopatológicos complementarios. Entre los posibles diagnósticos diferenciales, se debe tener en cuenta el órgano o sistema comprometido.

En el compromiso pulmonar, se deben considerar las micosis (mucormicosis, histoplasmosis, aspergilosis, blastomicosis, criptococosis), las infecciones por bacterias como *Actinomyces* o por micobacterias tuberculosas y no tuberculosas, y las neoplasias propias del pulmón.

En las manifestaciones cutáneas, la presentación clínica puede ser una guía diagnóstica. Si se presenta como celulitis, puede ser una infección causada por otros agentes como *Streptococcus pyogenes* o *Staphylococcus aureus*; si hay compromiso linfangítico, se plantea la posibilidad de esporotricosis o infecciones cutáneas por *Mycobacterium marinum*; y si son abscesos fríos, se debe contemplar la infección por *Actinomyces*, *Histoplasma* o *Cryptococcus*.

Dado que los abscesos son la presentación más común cuando hay compromiso del sistema nervioso central, es importante considerar un origen piógeno, y descartar otras condiciones como neoplasias malignas propias del sistema nervioso central, infartos vasculares, infecciones por agentes como *Toxoplasma gondii*, *M. tuberculosis*, hongos (como *Histoplasma* y *Cryptococcus*), y párasitos como *Taenia solium*, causante de la neurocisticercosis ^20^.

Las muestras clínicas que se pueden procesar en el laboratorio incluyen aquellas provenientes de secreciones respiratorias, abscesos, tejidos o biopsias de piel y líquido cefalorraquídeo, entre otras. Durante la observación de las muestras por microscopía, se deben buscar gránulos, más comunes en *N. brasiliensis*. El diagnóstico de laboratorio depende de la calidad de la muestra y su revisión exhaustiva, la técnica de fijación en parafina y la conservación de la muestra clínica en un tiempo de incubación extendido. Es clave alertar al profesional del laboratorio de microbiología sobre un potencial caso de nocardiosis, debido a que pueden ser necesarias técnicas y medios de tinción especiales para su diagnóstico.

**Figura 6:**
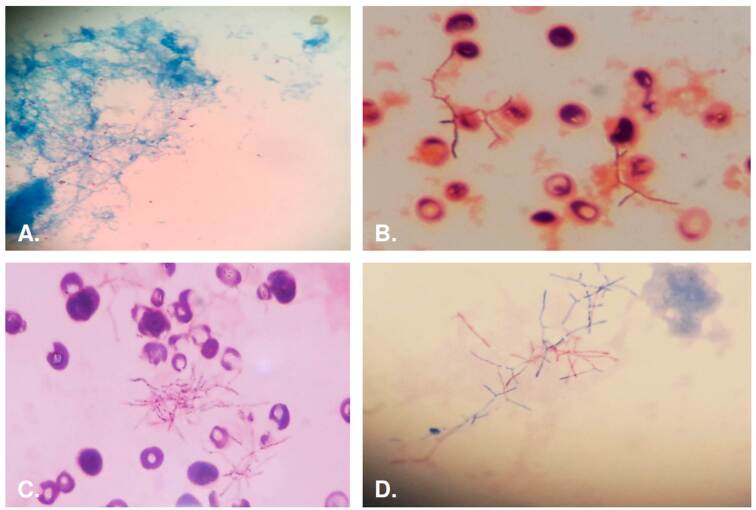
A. Caso 1. Se observan bacilos ácido-alcohol resistentes, más de 10 por campo en 20 campos observados. Ziehl-Nieelsen, 40X. B y C. Caso 4. Se observan bacilos grampositivos filamentosos en hemocultivo. Gram, 100X. D. Caso 4. Se observan bacilos parcialmente ácido-alcohol resistentes en hemocultivo. Zielh-Neelsen, 100X.

La tinción de Gram, la de Ziehl-Neelsen, el cultivo en medios no selectivos (agar-sangre y agar-chocolate) y los sistemas automatizados para el procesamiento de los hemocultivos, son útiles para identificar *Nocardia*, pues su crecimiento es lento y varía de 48 horas a semanas. En la tinción de Gram, *Nocardia* aparece como bacilos largos grampositivos, filamentosos (con ramificación en ángulo recto de diferentes tamaños (figura 6B y 6C). En caso de sospecha, se debe realizar una tinción modificada de Ziehl-Neelsen (figura 6A y 6D).

**Figura 7:**
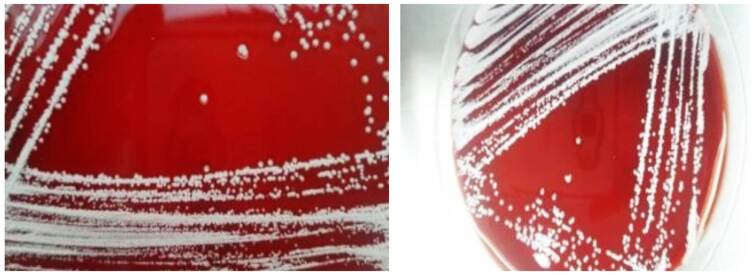
Caso 2. Cultivo de líquido pleural en medio de agar-sangre. Se observan colonias de color “blanco tiza” identificadas como *Nocardia* farscinica mediante MALDI-TOF.

La incubación del bacilo puede ser prolongada (hasta de tres semanas), aunque en la mayoría de los casos crece en tres a cinco días. Las colonias son secas y blanquecinas, lo que le da al cultivo un aspecto característico de tiza (figura 7 ). Sin embargo, la identificación microbiológica basada en aspectos bioquímicos o fenotípicos tiene grandes limitaciones. Las pruebas de identificación no convencionales se fundamentan en la biología molecular o la proteómica y utilizan tecnologías como MALDI-TOF, que permite una identificación precisa de la especie, como sucedió en los cinco casos de esta serie ^19^.

La especificidad de la MALDI-TOF en la identificación de especies ayuda a orientar el manejo antibiótico de la infección, dadas las limitaciones locales para practicar pruebas de sensibilidad *in vitro* y las dificultades técnicas, como la elección del método *in vitro*, la preparación del inóculo y la lectura e interpretación de los resultados.

Las únicas pautas internacionales disponibles son las del *Clinical and Laboratory Standards Institute* (CLSI). Según estas directrices, se debe realizar microdiluciones en caldo (BMD) para las pruebas de sensibilidad. Sin embargo, ningún estudio clínico ha demostrado que los desenlaces clínicos puedan predecirse a partir de los resultados obtenidos por esta técnica.

Además, puede no haber reproducibilidad entre laboratorios o presentarse una falsa resistencia (por ejemplo, para ceftriaxona e imipenem). Estos resultados se deben confirmar mediante otro método, como el cálculo de la concentración inhibitoria mínima utilizando tiras de prueba E o por difusión en disco de antibiótico en placas de agar. La prueba de sensibilidad de *Nocardia* a los antibióticos es compleja y requiere la comparación entre los datos *in vitro* y las sensibilidades esperadas a los antibióticos, basadas en la identificación de especies. Las diferencias más llamativas entre las especies de *Nocardia* son sus diferentes perfiles de sensibilidad a los antibióticos β-lactámicos. Por ejemplo, *N. cyriacigeorgica* es frecuentemente sensible a cefotaxima o ceftriaxona, mientras que *N. farcinica* es normalmente resistente a estos antibióticos. El imipenem parece ser el carbapenémico que más frecuentemente tiene actividad in vitro contra la mayoría de los aislamientos del complejo *N. nova*, y de las especies *N. cyriacigeorgica* y *N. farcinica*^21^.

En la histopatología de la biopsia, se pueden observar necrosis con microabscesos; los bacilos filamentosos (ramificados) se pueden visualizar mediante coloraciones de plata o Grocott ^1^. Traxler *et al*. indicaron que la biblioteca Bruker MBT Compass contenía 89 especies de *Nocardia*, mientras que la versión 3.2.0 de Vitek™ MS contenía solo 16 especies de *Nocardia*, las menos prevalentes. Cerca de 59 especies de la biblioteca de Bruker MBT) son difíciles de identificar con MALDI-TOF ^22^. Sin embargo, la identificación molecular rápida de *Nocardia* spp. se puede hacer por PCR, sondas de ADN, secuenciación, pirosecuenciación, ribotipado y análisis de polimorfismos de longitud de fragmentos de restricción ^1^.

La curva de disociación de alta resolución (*High-Resolution Melting*, HRM) de un producto amplificado por PCR en tiempo real, permite la detección de *Nocardia*, incluso en presencia de polimorfismos de un solo nucleótido. En un estudio realizado por Xu *et al*.^23^, se comparó la HRM con MALDI-TOF MS para la identificación de *N. farcinica*, *N. cyriacigeorgica* y *N. beijingensis*. Los resultados demostraron que MALDI-TOF MS identificó la especie del 94,1 % (80 de 85) de los aislamientos de *Nocardia*, mientras que la HRM identificó con precisión el 100 % (85 de 85) de los aislamientos. Además, la HRM demostró una mayor resolución para diferenciar los aislamientos de *N. beijingensis*, lo que indica que esta metodología es una excelente alternativa al MALDI-TOF.

La importancia de identificar la especie radica en su relación con el pronóstico de la enfermedad, la presentación de algunas manifestaciones clínicas o el potencial de invasión y diseminación. Se ha reportado que *N. farcinica* está más asociada con compromiso cerebral, cutáneo y de tejidos blandos, en comparación con otras especies. Lo anterior puede deberse a sus genes de virulencia, modificaciones de su pared celular y resistencia a especies reactivas de oxígeno, factores que contribuyen a peores desenlaces clínicos ^24^.

Los estudios de imágenes, como radiografías, tomografías computarizadas y resonancias magnéticas, ayudan a localizar anatómicamente las lesiones y establecer su tamaño. En caso de compromiso pulmonar, el hallazgo más común en la tomografía computarizada es el nódulo con cavitación o sin ella ^1^.

Teniendo en cuenta que hasta un tercio de los casos de nocardiosis presenta compromiso cerebral, la resonancia magnética tiene mejor rendimiento que la tomografía computarizada para identificar abscesos cerebrales ^25^. Sin embargo, hay dos estudios diagnósticos relevantes para la identificación de infecciones metastásicas: la tomografía por emisión de positrones con 18-fluorodesoxiglucosa combinada con tomografía computarizada (FDG-PET/ CT), y la espectroscopia por resonancia magnética de cerebro. Además de identificar focos de infección metastásica, la FDG-PET/CT permite establecer un enfoque terapéutico integral que incluye el abordaje quirúrgico y la duración del tratamiento; además, incrementa en un 57 % la posibilidad de identificar infecciones extrapulmonares previamente inadvertidas, lo que resalta su utilidad en la detección de compromiso cardiaco e intestinal ^26^.

Respecto al papel de la espectroscopia por resonancia magnética cerebral, esta técnica combina imágenes anatómicas con análisis bioquímicos para evaluar una región de interés. Permite discriminar lesiones neoplásicas de infecciones de tipo absceso cerebral, mediante las concentraciones de metabolitos como succinato, lactato o acetato, o por la disminución del índice colina/creatina. Hasta la fecha, la espectroscopia por resonancia magnética no permite identificar con precisión abscesos causados por *Nocardia*^27^.

Cuando en un subgrupo de pacientes no existe una causa clara de inmunocompromiso, es clave abordar de manera integral el diagnóstico, con énfasis en la identificación de la causa y, cuando sea posible, el tratamiento de las causas subyacentes. Cabe anotar que no hay un protocolo estandarizado para esta estrategia, por eso, se debe hacer una evaluación dirigida de los siguientes factores.

Los más frecuentes son:

- Indagar sobre el uso de esteroides, traumas o consumo de alcohol.
- Evaluar la posibilidad de diabetes mellitus mediante la medición de glucosa sérica en ayunas y posprandial.
- Identificar enfermedad pulmonar obstructiva crónica, exposición de humo de leña o tabaquismo.
- Establecer si hay alguna neoplasia hematológica mediante un hemograma o dado el caso, por análisis de medula ósea.
- Detectar si hay anticuerpos contra HIV-1 y 2.
- Averiguar si recibe alguna terapia inmunosupresora.

Los menos frecuentes son:

- Establecer si hay enfermedad granulomatosa crónica, que se debe sospechar en caso de infecciones recurrentes o por antecedentes familiares
- Descartar un síndrome de secreción de corticotropina (*adrenocorticotropic hormone*, ACTH) ectópica o síndrome de Cushing ectópico, cuando exista un hipercortisolismo no explicado

### Tratamiento

Para el tratamiento de la nocardiosis no hay estudios clínicos aleatorizados, ni guía de práctica clínica, por lo que la elección del tratamiento se hace según la zona geográfica y la especie identificada. Las pruebas de sensibilidad in vitro se recomiendan para todas las infecciones por *Nocardia*. Según el grado de sensibilidad in vitro, los aislamientos de *Nocardia* se clasifican en tres complejos mayores: *N. nova complex* (*N. nova*, *N. elegans*, *N. veterana*, *N. kruczakiae* y *N. africana*), *N. transvalensis complex* (*N. blacklockiae*, *N. wallacei* y Nocardia sp.), y *N. brevicatena*/*N. paucivorans complex*^1^.

La resistencia a los antimicrobianos está mediada por varios genes, como: *Tet* (K) para resistencia a tetraciclina; *mphA*, *mphB* y *mphC* para macrólidos; *blaTEM-1*, *blaSHV*, *blaZ*, *oxa* y *AmpC* contra β-lactámicos; genes *sul* para sulfonamidas; *aac*, *aph*, *ant*, *rmtA*, *rmtB*, *rmtC*, *rmtD* y *armA* contra aminoglucósidos; *gyrA* para fluoroquinolonas; y *23S-rRNA* y *cfr* para linezolid ^1^.

Desde hace más de 50 años se ha recomendado el tratamiento con trimetoprim-sulfametoxazol porque es eficaz contra la mayoría de las especies de *Nocardia*. La elección del tipo de tratamiento y su duración dependen de la localización, la extensión de la enfermedad y el estado del sistema inmunológico del paciente ^5^. En pacientes inmunocomprometidos, con enfermedad diseminada y compromiso del sistema nervioso central, se recomienda iniciar un tratamiento combinado con dos o más medicamentos, durante 6 a 12 meses. Entre las opciones de medicamentos activos que se pueden combinar, están: imipenem, ceftriaxona, quinolonas, linezolid, tetraciclinas y amikacina ^16^.

Los pacientes inmunocomprometidos tienen un mayor riesgo de desarrollar infecciones graves y, por eso, requieren de un tratamiento prolongado. El esquema terapéutico inicial en diez de los pacientes de esta serie, consistió en trimetoprim-sulfametoxazol, en seis el tratamiento fue combinado con carbapenem. A menudo se sugiere evitar la monoterapia con trimetoprim-sulfametoxazol en pacientes inmunocomprometidos ^13^.

Según las últimas recomendaciones para tratar infecciones por *Nocardia* en pacientes con trasplante de órgano sólido y en caso de afectación cerebral o enfermedad diseminada, la American Society of Transplantation indica, como tratamiento empírico, el uso de trimetoprim-sulfametoxazol asociado con un segundo agente (cefalosporinas de tercera generación, imipenem o amikacina) ^28^.

Un tratamiento de seis meses es apropiado para la mayoría de los casos ^5^. Sin embargo, en otros informes, la duración se establece según la localización de la infección. Por ejemplo, las infecciones pulmonares y de tejidos blandos se tratan durante al menos seis meses, mientras que la enfermedad diseminada requiere un tratamiento de seis a doce meses ^7^. Es importante hacer evaluaciones periódicas de la reacción al tratamiento y considerar los resultados de las pruebas de sensibilidad. En este estudio, se administraron tratamientos mucho más prolongados, incluso de 64 semanas. No se encontró indicación de profilaxis secundaria.

La tasa de mortalidad por infecciones causadas por *Nocardia* está alrededor del 20 al 50 % ^3^, pero puede variar dependiendo de varios factores, como la especie de *Nocardia*, la ubicación y la gravedad de la infección, el estado inmunológico del paciente y la oportunidad del tratamiento. En el presente estudio, hubo cuatro muertes en total; tres por cáncer y uno con antecedentes de cáncer basocelular de piel, pero con inmunosupresión proteico-calórica.

Entre las limitaciones de este estudio, se encuentra la escasa cantidad de casos reportados en más de 15 años de seguimiento en Colombia. Es probable que existan más casos no publicados. No obstante, podría haber un subregistro debido a que la nocardiosis no es una enfermedad de notificación obligatoria y a que, desde el punto de vista clínico, se puede confundir con otras causas.

Cabe resaltar que los casos aquí informados fueron detectados a partir de los resultados microbiológicos del laboratorio clínico de cada una de las instituciones y no por una búsqueda sistemática del código CIE-10 (Clasificación Internacional de Enfermedades). Se destaca la dificultad para identificar la especie en cada uno de los aislamientos. No obstante, esta situación no se presentó en todas las instituciones donde fueron tratados los pacientes de esta serie. Además, tampoco se contó con las pruebas de sensibilidad mediante sistemas automatizados ^9^, ya que las técnicas de biología molecular o espectrometría de masas no están disponibles en todas las instituciones de salud colombianas.

Por otra parte, aunque este sea un estudio retrospectivo que incluye varias instituciones de salud, los casos de nocardiosis se restringen a reportes de Bogotá, lo que limita la generalización de los resultados aquí presentados. Sin embargo, en la literatura colombiana relacionada, solo se encontraron reportes y series de casos más pequeñas que esta ^29^^,^^30^. No hay datos epidemiológicos más sólidos sobre esta infección en el país. La serie con mayor número de casos colombianos se publicó en 1986 ^29^ y documentaba 10 casos. Por lo tanto, en el presente estudio, se registra el mayor número de casos de nocardiosis informados hasta el momento.

La importancia de los casos aquí descritos radica en que la nocardiosis es una infección que se documenta en pacientes con inmunosupresión, ya sea por cáncer de órgano sólido o neoplasias malignas hematológicas, HIV o consumo de medicamentos. Actualmente, la infección es “más común” debido a los métodos diagnósticos modernos que implementan tecnologías avanzadas para una detección más oportuna.

En la nocardiosis, el compromiso de más de un órgano exige extender los estudios de imagen al sistema nervioso central; en esta localización, se requiere una terapia antimicrobiana combinada, con antibióticos capaces de alcanzar concentraciones adecuadas en dicho sistema. En los pacientes evaluados, la especie identificada con mayor frecuencia fue *N. farcinica*. *In vitro*, esta especie es sensible a trimetropim-sulfametoxazol y, por esta razón, tal compuesto sigue siendo el tratamiento empírico de elección. No obstante, en la actualidad, se sugieren esquemas terapéuticos con antibióticos combinados para la nocardiosis diseminada. Los diagnósticos diferenciales son variados, e incluyen infecciones por *M. tuberculosis*, actinomicetos y hongos. La sospecha clínica y la tinción de Gram permiten el inicio rápido del tratamiento, lo cual impacta positivamente en la supervivencia de los pacientes.
